# Assessing demand-side barriers to uptake of intermittent preventive treatment for malaria in pregnancy: a qualitative study in two regions of Uganda

**DOI:** 10.1186/s12936-016-1589-7

**Published:** 2016-11-04

**Authors:** Christian Rassi, Kirstie Graham, Rebecca King, James Ssekitooleko, Patrobas Mufubenga, Sam Siduda Gudoi

**Affiliations:** 1 Malaria Consortium, Development House, 56-64 Leonard Street, London, UK; 2 Nuffield Centre for International Health and Development, Leeds Institute of Health SciencesUniversity of Leeds, Leeds, UK; 3 Malaria Consortium Uganda, Plot 25 Upper Naguru East Road, Kampala, Uganda; 4 Malaria Consortium Uganda and PAMU Consults (U) Ltd, Plot 577, Block 15, Nsambya Road, Kampala, Uganda

**Keywords:** Malaria in pregnancy, Prevention, IPTp, Intermittent presumptive treatment, Intermittent preventive treatment, Antenatal care, ANC, Coverage, Uptake, Operational research, Malaria Consortium, COMDIS-HSD

## Abstract

**Background:**

To prevent malaria infection during pregnancy in endemic areas in Africa, the World Health Organization recommends the administration of intermittent preventive treatment in pregnancy (IPTp) as part of the focused antenatal care package. However, IPTp uptake in most countries remains low despite generally high antenatal care coverage and increased efforts by governments to address known bottlenecks such as drug stock-outs. The study explored factors that continue to impede uptake of IPTp among women who attend antenatal care. This paper focuses on demand-side barriers with regard to accessibility, affordability and acceptability.

**Methods:**

The research was conducted in 2013/2014 and involved 46 in-depth interviews with four types of respondents: (i) seven district health officials; (ii) 15 health workers; (iii) 19 women who attended antenatal care; (iv) five opinion leaders. Interviews were conducted in Eastern and West Nile regions of Uganda. Data was analysed by thematic analysis.

**Results:**

District health officials and health workers cited a range of barriers relating to knowledge and attitudes among pregnant women, including lack of awareness of pregnancy-related health risks, a tendency to initiate antenatal care late, reluctance to take medication and concerns about side effects of IPTp. However, women and opinion leaders expressed very positive views of antenatal care and IPTp. They also reported that the burden of travel and cost associated with antenatal care attendance was challenging, but did not keep them from accessing a service they perceived as beneficial. The role of trust in health workers’ expertise was highlighted by all respondents and it was reported that women will typically accept IPTp if encouraged by a health worker.

**Conclusions:**

Given the positive views of antenatal care and IPTp, high antenatal care coverage and reported low refusal rates for IPTp, supply-side issues are likely to account for the majority of missed opportunities for the provision of IPTp when women attend antenatal care. However, to increase uptake of IPTp on the demand side, health workers should be encouraged to reassure eligible women that IPTp is safe.

**Electronic supplementary material:**

The online version of this article (doi:10.1186/s12936-016-1589-7) contains supplementary material, which is available to authorized users.

## Background

Pregnant women are more susceptible to malaria infection than non-pregnant women and have a higher risk of suffering from severe disease. In areas of high and stable malaria transmission, where women have acquired high levels of immunity, malaria infection during pregnancy is often asymptomatic, but can still have devastating consequences for both mother and child, including maternal anaemia, stillbirth, and low birth weight, a major risk factor for infant mortality and morbidity [1, 2].

Preventing and controlling malaria infection during pregnancy is therefore an important strategy to improve maternal and newborn health. In areas of high and moderate transmission in Africa, the World Health Organization (WHO) recommends a three-pronged approach [3]:

1. Effective case management for malaria illness and anaemia during pregnancy;
2. The use of insecticide-treated nets by pregnant women;
3. Intermittent preventive treatment in pregnancy (IPTp), which involves repeated administration of curative doses of a safe and effective anti-malarial to pregnant women, without testing the recipient’s infection status. The drug used for IPTp is called sulfadoxine-pyrimethamine (SP) and is also known under its brand name Fansidar^®^. Pregnant women typically receive IPTp as part of the focused antenatal care (ANC) package, which specifies that pregnant women should have at least four assessments at specified intervals by or under the supervision of a skilled attendant [4]. WHO currently recommends IPTp at each ANC visit after the first trimester, with doses given at least 1 month apart [5]. IPTp should be administered as directly observed therapy (DOT).

Even though ANC coverage in African countries is generally high, many countries have struggled to achieve high levels of uptake of IPTp [6], which suggests that opportunities for the provision of IPTp during ANC are being missed. It should be noted, however, that there are a number of complicating factors:

- Not all pregnant women attending ANC are eligible for IPTp. It should not be provided to women in the first trimester or to women who are human immunodeficiency virus (HIV) positive and taking co-trimoxazole prophylaxis.
- Women do not necessarily follow the recommended four-visit ANC schedule. If they initiate ANC late during their pregnancy or visit infrequently, there will be limited opportunities for provision of subsequent doses of IPTp. Attendance for the third or fourth visit is typically much lower than the first and second visit.

Governments and implementing partners in many countries have stepped up efforts to increase uptake of IPTp, addressing some of the known bottlenecks, such as stock-outs of drugs and commodities at ANC clinics and lack of guidance and supervision for health workers [7, 8]. However, IPTp coverage remains low in many countries where malaria is endemic [9].

While there is a growing body of quantitative data exploring barriers to and determinants of IPTp uptake, comparatively few studies have sought to approach the issue from a qualitative point of view [7, 10]. There is hence limited understanding of the barriers that continue to impede IPTp despite high ANC attendance and increased attention on the part of governments and implementing partners. This study explored thoughts and opinions of key stakeholders (district health officials, health workers, pregnant women and mothers, opinion leaders) to better understand how stakeholders’ perceptions may affect uptake of IPTp, particularly for those women who attend ANC, albeit not necessarily following the recommended four-visit schedule. The study therefore sought to address two research questions:

1. What are the barriers to women receiving or taking IPTp during ANC?
2. What are the barriers to women initiating ANC early and attending repeatedly?

The study was conducted with the aim of informing the development of an intervention that could help increase uptake of IPTp for women attending ANC. Given the volume of data collected and large number of topics explored, data relating to demand and supply-side issues are presented in separate papers. While acknowledging that supply and demand side are inevitably interconnected and that under programmatic conditions it is typically necessary to address both sides concurrently, the authors nevertheless found applying an analytical framework which uses this distinction as its starting point useful in exploring the multitude of factors affecting utilization of health services [11]. This paper presents data relating to demand-side barriers, specifically challenges relating to accessibility, affordability and acceptability of ANC and IPTp. Note that this includes data from respondents representing both the supply (health workers, health officials) and the demand side (women, opinion leaders). Results relating to supply-side issues have been published elsewhere [12].

## Methods

### Study design

The study was a cross-sectional qualitative study, which involved a total of 46 in-depth interviews with four types of respondents: district health officials, health workers, women who had attended ANC and opinion leaders (for example traditional birth attendants, local councillors and teachers). Sample size and approach were determined taking into account available time and budget to allow for a thorough, in-depth exploration of the research questions from the point of view of a range of key informants. Reporting of the study methods and results follows the consolidated criteria for reporting qualitative research (COREQ) [13]. The study also included a document and record review at four health facilities. However, as the findings from this review relate exclusively to supply-side barriers, they are not reported in this paper.

### Study setting

#### IPTp and ANC in Uganda

In Uganda, country-wide implementation of IPTp with SP started in 2001. According to the most recent household survey data available at the time of the research, only a quarter of women received at least two doses of IPTp in 2011, while 90% attended ANC at least twice and 48% followed the recommended schedule of four ANC visits. The median month of gestation at which women initiated ANC was 5.1 [14]. According to the latest available household survey data at the time of publication, uptake of two or more doses of IPTp had increased to 45% in 2014/15 [15].

At the time of data collection, the country had not yet adopted the latest WHO policy recommendation of monthly administration of SP. The different policy documents and guidelines governing IPTp provision at the time were “inconsistent or unclear” [16], but generally suggested that women should receive a maximum of two doses of SP during the course of a pregnancy. IPTp is provided as part of the ANC package at health centres II, III, IV and hospitals. ANC services should be provided free-of-charge to women accessing ANC through public health facilities.

### Study regions, districts and health facilities

**Fig. 1:**
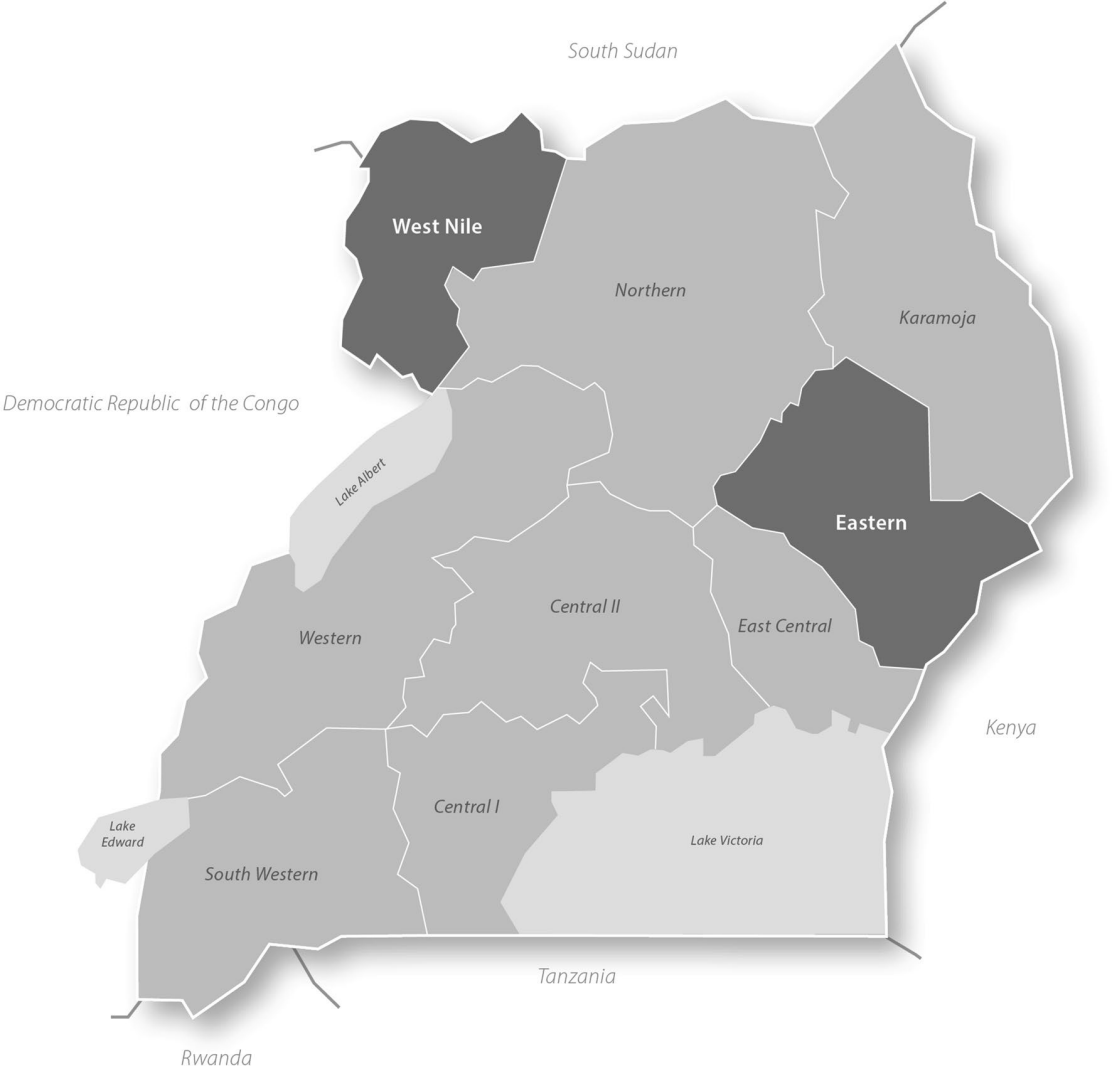
Map of the Republic of Uganda. Study regions highlighted in *dark grey*

The study was conducted in two regions of Uganda: Eastern and West Nile (see Fig. 1). Eastern region has historically received high levels of support from implementing partners and has consistently been among the regions reporting higher than average IPTp uptake. West Nile, on the other hand, has received less investment and typically reports IPTp uptake levels below the national average. According to the most recent household survey data available at the time of the research, 33 and 21% of women in Eastern and West Nile regions received two doses of IPTp respectively in 2011. In 2014/15, this figure was 36% for Eastern and 35% for West Nile [15]. Table 1 presents ANC and IPTp uptake data from Uganda’s Health Management Information System (HMIS) for the four study districts compared with the national and regional average for the 2 years preceding data collection (2011–2012). Note that the regional uptake patterns found by household surveys are not consistently reflected in the HMIS data, which are compiled from health facility reports. The HMIS data was extracted for this publication and not available to the study team at the time of selecting the study regions and districts.

**Table 1:** Antenatal care and IPTp uptake data (%) in study districts, 2011–2012 (Source: Health Management Information System, Republic of Uganda)

	ANC1^a^		ANC4^b^		IPT1^c^		IPT2^d^	
	2011	2012	2011	2012	2011	2012	2011	2012
Uganda	90	84	29	28	58	79	44	50
Eastern	73	67	18	18	79	83	51	52
Urban study district	57	66	15	29	97	96	70	76
Rural study district	43	61	6	9	78	93	66	55
West Nile	46	57	18	22	83	86	59	62
Urban study district	61	101	19	36	76	81	50	54
Rural study district	20	22	13	11	92	92	71	69

^a^ANC1: First antenatal care visit; denominator: expected pregnancies (5% of total population in the area)

^b^ANC4: fourth antenatal care visit; denominator: expected pregnancies (5% of total population in the area)

^c^IPT1: first dose of IPTp; denominator: ANC1

^d^IPT2: second dose of IPTp; denominator: ANC1

**Fig. 2:**
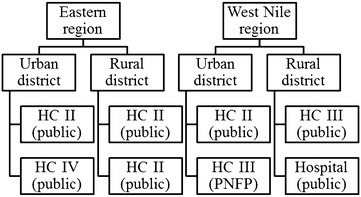
Types of health facilities sampled. *HC* health centre, *PNFP* private not-for-profit

Within each region, the study team selected one rural and one urban district based on convenience. Two health facilities were selected in each district, again by convenience sampling, but taking care to include the different levels of health facilities involved in providing ANC, as well as public and private not-for-profit (PNFP) providers. Private for-profit facilities were excluded from the sample as the Ministry of Health in Uganda does not provide specific support on IPTp to this type of facility. Figure 2 shows the types and levels of health facilities sampled in each study district.

### Participant selection

#### District health officials

Two district health officials were purposively selected in each of the four study districts with the help of the District Health Officer. The selection was based on their involvement in malaria or maternal health programming, as well as health supervision. In one district, only one suitable interviewee was available on the day of the research team’s visit, so a total of seven interviews with district health officials were conducted. These included three Malaria Focal Persons, one Assistant District Health Officer in charge of maternal and child health, one Health Educator, one Stores Assistant and one HMIS Focal Person.

### Health workers

Two health workers involved in the provision of ANC or supervision of ANC services were purposively identified at each of the eight health facilities with the help of the facility in-charge. At one facility only one suitable interviewee was available on the day of the research team’s visit, so a total of 15 interviews with health workers were conducted—seven with the most senior member of staff present and eight with midwives.

### Women

At each of the eight health facilities, two women who had accessed ANC and lived within the catchment area were identified with assistance from health workers. Generally, one of the women selected was pregnant at the time of the interview, while the other had given birth during the previous 12 months. Similarly, where possible, the team selected one woman who lived within walking distance from the facility and one woman from the fringes of the facility’s catchment area, in order to determine whether distance from the facility affected her perceptions of IPTp. However, it proved difficult to apply distance from the health facility as a sampling criterion and relevant information was not consistently captured in the field.

The initial analysis of data collected from these 16 women using health workers as gatekeepers showed that all women had received a full course of IPTp and had generally positive views of ANC and IPTp. The research team felt that additional data should be collected from women who had not received a full course of IPTp, defined as at least two doses as per national guidelines at the time. A second round of data collection was therefore conducted, where women were identified from facilities’ ANC registers without involvement from health workers. A total of 12 women who, according to the register, had not received IPTp when they visited ANC, were identified. However, only three additional interviews were conducted (all with women who had recently given birth). The remaining nine women identified indicated that they had in fact received IPTp at that visit, and it had therefore been incorrectly recorded in the register, or they had been informed that they were not eligible for IPTp when they visited ANC, so had correctly not received it. Two of the three women interviewed in round two stated that they had received one dose of IPTp at a previous visit. A total of 19 women were interviewed during two rounds of data collection. Though the sample size is not large enough to draw valid conclusions with regard to the accuracy of uptake figures, the research team’s difficulties in finding women who had not received a full course of IPTp may suggest higher than expected uptake compared with those reported through household surveys or HMIS. Data accuracy has been highlighted as a challenge to effective monitoring and evaluation of malaria interventions by the Government of Uganda [17], a conclusion which is also supported by supply-side findings from this study [12].

### Opinion leaders

To test the largely positive views expressed by women about IPTp and ANC during the first round of data collection, five opinion leaders who were considered influential in shaping communities’ perceptions of maternal and child health were identified with the help of the sub-county Secretary for Health. This included two traditional birth attendants, one community councillor, one teacher and one retired midwife.

### Data collection

#### Field work and researcher training

CR and eight local field researchers (five female, three male) conducted the first round of data collection in November 2013. All field researchers had relevant language skills and were university or college graduates with previous experience of mainly quantitative field research. Prior to conducting the field work, they attended a five-day training conducted by CR, which covered the study design, information on malaria in pregnancy and IPTp, as well as qualitative research techniques and best practice. The second round of data collection was conducted in April/May 2014 by CR and three of the field researchers (one female, two male) who had been involved in the first round of data collection. Field researchers involved in round two received a 1-day refresher training conducted by CR. Interviews generally lasted between 45 and 75 min. While in the field, researchers had daily briefings with CR to discuss and resolve challenges.

### Interview guides

Guides for semi-structured interviews with district health officials, health workers and women were developed in English by CR, KG, RK and SGS before the first round of data collection (see Additional files 1, 2, 3). The interview guide for opinion leaders was developed by CR and KG before the second round of data collection (see Additional file 4). Interview topics explored were based on a framework of themes derived from a review of the literature on barriers to IPTp uptake, as well as authors’ experience of implementing malaria in pregnancy interventions. See Table 2 for an overview of topics relating to demand-side issues explored in the interview guides by type of respondent. All interview guides included open-ended general questions about interviewees’ perceptions of IPTp and the reasons behind the low uptake of the service. This enabled respondents to also bring up topics not specifically covered by the interview guides.

**Table 2:** Demand-side topics covered by interview guides by type of respondent

Topic	District health officials	Health workers	Women	Opinion leaders
Burden of travel	–	–	X	–
User fees	–	X	X	–
Indirect costs	–	–	X	–
Vulnerability and social support	–	–	X	X
Formal vs. traditional care	–	–	X	–
Malaria and malaria prevention	–	–	X	X
Timing and frequency of ANC attendance	–	X	X	–
Attitudes towards ANC	–	–	X	X
Attitudes towards IPTp and SP	–	X	X	X

X denotes that this topic was covered by a specific question or probe in the interview guide

Interview guides were discussed in detail during the researcher training. The interview guide for women was translated into the local languages spoken in the study districts (Ateso, Lugbara and Madi) by researchers who were native speakers of those languages. Accuracy of the translation was checked by field researchers with native language skills in the respective languages during the training preceding the field work. The interview guides for district health officials, health workers and women were subsequently pre-tested with relevant participants from a district that was not included in the study. As the interview guide for opinion leaders was based on questions used with other types of respondents, pre-testing was not considered necessary. Table 3 provides an overview of the number of interviews conducted, how interviewees were approached, where interviews were conducted and which language was used with the different types of respondents.

**Table 3:** Number of interviews conducted, approach, location and language used by type of respondent

	District health officials	Health workers	Women	Opinion leaders
No of interviews	7	15	19	5
Approach	By District Health Officer about 1 week before the researchers’ visit to the district	Facility in-charge about 1 week before the researchers’ visit to the health facility	By researchers on the day of the interview	By researchers on the day of the interview
Location	District health office	Respondent’s place of work	Respondent’s home	Respondent’s home
Language	English	English	Local language	English

### Transcription and translation

All interviews were audio-recorded. Those conducted in English were transcribed verbatim and those conducted in a local language were translated and transcribed in English directly from the recording. All were transcribed by the researcher who had conducted the interview. Field researchers were required to take notes during the interview and to include contextual information (e.g. non-verbal clues) in the transcripts.

### Data analysis

Interview transcripts were managed using NVivo 10 (QSR International) qualitative analysis software. Thematic analysis, using a combination of deductive and inductive approaches, was carried out by CR, KG and RK, who agreed an initial coding frame based on the framework used for the development of interview guides and analysis of a sample of five transcripts. All transcripts were coded by CR who discussed and agreed subsequent changes to the coding frame with KG and RK, taking into account emerging themes. The headings used in the results and discussion section of this paper reflect the main codes used for the analysis of demand-side issues.

## Results and discussion

Demand-side barriers to IPTp uptake will be discussed under the themes of accessibility, affordability and acceptability.

### Accessibility

District health officials and health workers often speculated that living at a distance from the nearest health facility and the effort required in travelling to the point of care kept some pregnant women from accessing ANC or from attending ANC early and consistently. Several of the women interviewed who lived in more remote communities confirmed that travelling to ANC, and in particular having to walk or ride on motorcycles for long distances and along poor roads, was burdensome:

Woman 5: *If your body becomes too heavy, walking also becomes difficult if there is nobody to bring you.*

The statement may also reflect a reluctance to travel far during the later stages of pregnancy. However, none of the women interviewed suggested that the burden of travel had influenced their decision to attend ANC. Several respondents explicitly stated that they felt it was important to attend ANC despite this challenge:

Researcher: *Did the effort involved in travelling to the facility influence when and how often you visited a doctor or midwife for check*-*up?*

Woman 18: *No, because I felt I must take care of myself and the baby to ensure that both of us are healthy.*

This suggests that distance to the point of care and lack of transport, which have been cited by studies conducted in a range of African countries [18, 19] are not likely to act as barriers to repeated ANC attendance and IPTp uptake among women who generally attend ANC. The concerns over the burden of travel raised by some of the women interviewed who lived at a distance from the nearest health facility, though they did not keep the respondents from attending ANC, suggest that having to travel long distances may act as a disincentive for women who do not attend ANC at all.

### Affordability

The majority of women and community leaders confirmed that all ANC services at public facilities were offered free of charge in line with government policy. However, several women in West Nile who attended ANC at public facilities reported they had been asked to pay a small fee when they first visited ANC or they had been required to pay for an ANC card:

Woman 14: *I spend the money only for buying antenatal card. And I don’t know, but during my first visit they told me to pay 500 shillings.*

While private for-profit facilities are expected to procure SP, the government has committed to providing SP free-of-charge to public facilities. It also provides funds to PNFP facilities to procure essential medicines, including SP. However, this study found that stock-outs appear to persist in PNFP facilities [12]. Health workers at the PNFP facility included in the study sample indicated that they may charge women for the provision of IPTp or ask them to buy SP and bring it to the facility when they are experiencing stock-outs of the drug:

Researcher: *Are women required to pay for the provision of IPTp at your facility?*

Health worker 13: *Yes.*

Researcher: *How much?*

Health worker 13: *One thousand Ugandan shillings when [we] buy [it] ourselves. Due to stock*-*out, we ask money from them at no profit, but they don’t pay when available.*

It can therefore be assumed that women accessing ANC through private providers are likely to incur costs for the provision of IPTp, at least occasionally.

In addition to direct costs, several of the women interviewed, in particular those who lived at a distance from the nearest health facility, referred to the indirect costs of visiting ANC, for example costs associated with travelling to the health centre or paying for accompanying relatives. The opportunity costs associated with ANC attendance were also occasionally mentioned:

Woman 6: *And when you reach here* [the health facility], *you are tired and yet you have to go back and you won’t be able to do any work, so that means you have to over think whether to come.*

This issue was also raised by a number of district health officials and health workers in West Nile, who speculated that some women may not be able to attend ANC regularly because of what they perceived as cultural pressure on women to contribute towards their families’ livelihood:

District health official 6: *So* – *and then our social works. Women* – *by the role of the culture here* – *women are exposed to too much work. So you find a pregnant mother* – *she’s busy in the garden and what. So she does not come for* – *to attend to the antenatal, where she can get this treatment.*

About one third of the women interviewed stated that, overall, the costs associated with attending ANC were low and did not present a challenge to ANC attendance. However, the majority reported that given the widespread poverty and lack of financial support from partners and families, they found the financial burden of ANC attendance challenging:

Researcher: *Was it difficult to find the money to pay for the visit?*

Woman 17: *Yes and being the only child and no support from any one.*

None of the women interviewed indicated that cost had affected their decision to attend, however. Several stated that they felt it was important to make use of the service despite the financial burden and described how they had to be resourceful and plan ahead in order to ensure the financial means were available when needed:

Woman 3: *To get money I go to some garden to work and they give you from there [inaudible], then I come and go to the hospital. You have to estimate that* – *tomorrow I have to go, so you start today looking for where there is any casual work. If someone says that you come and help me and gives you money, tomorrow you go to the hospital.*

Both direct and indirect costs, real or perceived, have been discussed as barriers to uptake of ANC and IPTp in the literature [20, 21]. While the findings from this study suggests that affordability is not likely to be a major barrier among those who generally attend ANC and those who attend in the public sector, the impact of user fees on women who do not attend ANC and those who attend at private sector facilities should be investigated in more detail. In general, user fees levied at private facilities have been associated with the emergence of an equity gap in terms of access to health care in Uganda, with the poorest unlikely to afford the services [22]. This is particularly relevant because of the considerable size of the private health care sector in the country [23].

### Acceptability

#### Vulnerability and social support

Most women and all community leaders acknowledged that pregnant women are more vulnerable compared with non-pregnant adults. They mentioned a range of pregnancy-related health issues and emphasized that pregnant women need to adapt behaviour patterns due to their heightened vulnerability during pregnancy, for example eating a healthy diet and paying more attention to hygiene and cleanliness. Many reported that their partners, family and community leaders take a special interest in their wellbeing while they are pregnant.

However, several women complained that their partners did not appreciate women’s heightened vulnerability during pregnancy:

Woman 15: *If my husband forces me to do work and yet am sick, I cannot do the work as he wanted. He will even want to fight me that will now force me to go and explain to my brother*-*in*-*laws.*

Researcher: *Does he fight you while you are pregnant?*

Woman 15: *Yes.*

Lack of emotional and practical support from partners was also highlighted as a potential barrier for ANC attendance by a range of district health officials and health workers. However, while involving partners in ANC has been found to generally improve health services for mother and child [24] and should therefore be encouraged, based on the findings from this study it appears unlikely that this will improve IPTp uptake among women who generally attend ANC.

### Formal versus traditional care

All women and opinion leaders expressed very positive views with regard to seeking care from qualified providers, especially during pregnancy. Many emphasized the role of trust in health workers’ expertise:

Woman 1: *In case there is something disturbing my health, I will go to visit a doctor to tell my problem and we share. After, he advises me on what to do. Thus I can get peace of mind. Because when I am at home, I can’t know what problem or disease that disturbs me. Thus I have to go to the hospital for test to determine the real problem.*

Two district health officials in West Nile indicated that reservations with regard to formal care providers may persist in more remote communities. They speculated that women may prefer to use the services of traditional birth attendants, possibly because they are more responsive to women’s needs. This was confirmed by two women in West Nile who reported that some of their peers preferred traditional medicine. However, several of the women interviewed expressed a clear preference for formal care:

Researcher: *As an individual what do you know about the local medicine and what effects they have when taken?*

Woman 18: *The local medicines always have bad effects like causing abortion and even death and those giving them do not first examine you before administering it.*

As several studies have shown preference for traditional care over formal health care in sub-Saharan Africa [25, 26], the widespread preference for formal care reported in this study is an encouraging finding.

### Taking medication

Most district health officials and health workers hypothesized that pregnant women may not want to take medication and may therefore refuse to take IPTp. However, all women and opinion leaders expressed generally positive attitudes towards taking medication if it is beneficial to the wellbeing of mother or child. Many explicitly mentioned receiving medication as a benefit of seeking care from a qualified provider, in particular women from Eastern region, where the term used to refer to ANC in the local language literally translates as ‘taking medication’:

Woman 6: *The thing that made me come was to take medication. Because they call that ‘taking medication’.*

Many women stated that while they were generally hesitant to take medication while pregnant, they trusted nurses’ and doctors’ judgement and expertise and were prepared to accept medication when recommended by a health worker:

Woman 16: *Another one is not to use drugs unnecessary. If you are pregnant, you should use drug prescribed to you in the health facility. Don’t buy drugs from the vendor and swallow like that.*

Concerns over taking medication while pregnant have been discussed as a potential barrier to IPTp uptake in the literature [27]. However, it appears that most women can be persuaded to accept medication if the benefits are pointed out by a qualified health care provider. This confirms that women’s trust in health workers’ judgement plays a crucial role in ensuring high IPTp uptake, which has been highlighted by a number of previous studies [28–30].

### Malaria and malaria prevention

District health officials and health workers frequently mentioned that pregnant women’s and communities’ lack of awareness of the risks of malaria in pregnancy was likely to be one of the main reasons behind the low uptake of IPTp. However, all opinion leaders and women interviewed showed some level of risk awareness. For example, the majority of women stated that pregnant women were more susceptible to malaria than non-pregnant women and most knew that malaria infection during pregnancy can lead to miscarriage. Several women also knew that malaria in pregnancy can negatively affect the health of the child.

Women and opinion leaders generally agreed that it was important for pregnant women to take steps to avoid malaria infection. With one exception, all women reported sleeping under a mosquito net. Even though district health officials speculated that women may not believe that taking medication was an effective malaria prevention mechanism, all but one of the women interviewed were supportive of the idea of taking preventive medication, particularly when prescribed by a health worker:

Woman 12: *If the drugs are prescribed to you, then you have to accept, you should follow the instructions.*

This suggests that lack of risk awareness, which has frequently been cited as a barrier to IPTp uptake in the literature [31], does not contribute to missed opportunities for the provision of IPTp among women attending ANC, a finding supported by a study in Ghana, Kenya and Malawi, which concluded that a majority of women were aware of the risks of malaria in pregnancy [32]. It was evident, however, that many women and opinion leaders did not distinguish clearly between taking medication to prevent malaria and taking medication to treat symptomatic malaria, a challenge which has also been reported from Malawi and Ghana [33].

### Timing and frequency of ANC attendance

The most frequently cited challenge for repeated administration of IPTp by district health officials and health workers was that pregnant women tended to initiate ANC late during their pregnancy, which means there may not be sufficient time to provide more than the first dose of IPTp:

Health worker 2: *Through experience some of our mothers just stay at home. And then come in the third trimester, so you find that there is no way you can fix a second dose. The mother will either take a first dose and deliver or the mother may come in labour.*

Several district health officials and health workers in West Nile also stated that many pregnant women attended ANC infrequently, which would also lead to missed opportunities for the provision of repeated doses of IPTp:

District health official 7: *Ok* – *about the IPTs I think […]* – *you know mostly* – *mostly in Uganda, women just come for the first* – *first ANC. Ya. After first ANC, they sometimes disappear. They don’t come for the* – *the* – *those remaining one. So they miss the IPT.*

Some respondents speculated that women may attend ANC late or infrequently because they fail to see the need for continuous services, particularly once the pregnancy has been confirmed as generally uncomplicated. Another possible reason cited for late attendance was that women may only attend ANC in order to receive an ANC card, which entitles them to receive care from a skilled provider when they give birth. Only a few midwives acknowledged that while late and infrequent ANC attendance was a challenge in principle, the majority of women attended ANC regularly.

By contrast, all women interviewed, including those who had not received a full course of IPTp, were frequent and regular ANC attenders. Of those who had given birth at the time of the interview, all had attended ANC at least twice and most had followed the recommended four-visit schedule. There was some variation with regard to when women had initiated ANC, but most reported that they had first attended ANC between the third and fifth month of gestation. Most of the women and opinion leaders interviewed knew that the recommended number of ANC visits during the course of a pregnancy is four. Only two, both in West Nile, were not aware of the recommended four-visit schedule. A number of women emphasized the role of midwives in encouraging pregnant women to attend ANC frequently and reminding them to complete the recommended ANC schedule.

Health workers’ perception of late and infrequent ANC attendance as the main barriers to IPTp uptake have been highlighted by a recent study in Mali [34]. While late attendance has been cited as a barrier to IPTp uptake in the literature [35, 36], it seems likely that this no longer plays a significant role. This research and several other recent studies did not find any evidence of late ANC attendance or a significant effect of the timing of ANC visits on IPTp [37–39]. A study conducted in Uganda in 2008 found that the median gestational age at first ANC visit was 5.7 months with the vast majority of pregnant women returning for subsequent visits, leaving plenty of opportunities for the provision of at least two doses of IPTp [40].

### Attitudes towards ANC

All women, including those who did not receive a full course of IPTp, and all opinion leaders interviewed, expressed generally positive views with regard to attending ANC. Many made general comments about the role of ANC visits in ensuring the health of both mother and unborn child and in detecting pregnancy-related complications. The most frequently mentioned specific service provided as part of ANC was checking the baby’s position. Other benefits mentioned included receiving incentives such as mosquito nets, health education and the opportunity to get tested for HIV and other diseases. Several women and opinion leaders referred to receiving IPTp as one of the main benefits of attending ANC.

District health officials and health workers, on the other hand, suspected that negative or indifferent attitudes towards ANC persisted and played a role in women missing out on the provision of IPTp. Some speculated that this may be due to a generally defeatist attitude among women or a culturally motivated reluctance to disclose their pregnancy early. Several respondents suggested that pregnancy may not be perceived as a health issue and women may not see the need to visit a health facility if they are not sick:

District health official 1: *I think, eh, the women do* – *do not necessarily fall sick, so that one has in mind* – *I have to go in what?* – *The health unit. So for them, they take for granted* – *after all they are what? [They] are healthy, why do I need to go there?*

Many of the issues raised by district officials and health workers have been discussed in the literature. For example, reluctance to reveal pregnancy early on has been reported in Mali [34] and a tendency to only seek help if sick has been described in Mozambique [41]. While this was not confirmed by any of the women interviewed in this study, it was noticeable that many women tended to talk about seeking care and treatment when they are experiencing symptoms of sickness rather than to prevent sickness. Similarly, four women reported that they had initiated ANC because they felt unwell and were concerned about the baby’s health. It is therefore possible that a lack of awareness of the preventive benefits of ANC and IPTp may act as a barrier among those who do not attend ANC at all.

### Attitudes towards IPTp and SP

Women and opinion leaders were generally supportive of the concept of IPTp. The majority of women interviewed during the first round of data collection remembered that they had received and taken a drug called Fansidar^®^ when they attended ANC. Some could not remember the name, but were able to describe the drug and knew that it was used to prevent malaria. Women interviewed during the second round of data collection because they had not received a full course of IPTp stated that they had not been offered IPTp by the health worker and that they would have accepted it, had it been offered to them.

Many women, including those who had not received a full course of IPTp, stated that IPTp was effective in preventing the effects of malaria in pregnancy on mother and child. While some of the statements with regard to the benefits of IPTp did not reflect accurate biomedical knowledge, they illustrate the generally positive views held by respondents:

Woman 8: *It makes the baby healthy and when you give birth, the child will be good. But not the one with a big head, disabled* – *and not the child which has got funny looks. But you will give birth to a beautiful baby when you use this drug.*

The very positive perceptions of IPTp and willingness to take SP when offered was confirmed by the majority of health workers:

Health worker 3: *Actually, they* – *in our health facility, they don’t refuse. They always take when we give them. They take.*

Though none of the respondents expressed concerns over the safety of IPTp, all types of respondents suggested that fear of side effects may contribute to the low uptake:

District health official 6: *I happened to be in one of the health centre IV and I happened to be in* – *interacting in one of the community around. Basically, there is [inaudible], when I was interacting* – *the side effects of the drug, the Fansidar* – *see, when they take it, they find it difficult. That is one reason.*

While most of the women interviewed had not experienced unpleasant side effects (including the two women interviewed during the second round of data collection who had previously received the first dose of IPTp), five reported that they had felt dizzy or nauseous after taking the medication. Some also reported that they had heard complaints of side effects from friends and family:

Woman 1: *Personally I have never seen any effect, but my sisters and other pregnant women have complained of this drug. For example, some say it has given my body to itch, swelling, pain for so long. Others say it makes me to vomit* – *dizziness is what I have heard.*

There appeared to be a particular concern about taking SP on an empty stomach. This was widely reported by all types of respondents:

Health worker 6: *The challenge I have is mostly on the mothers. Because most of them don’t* – *they feel like they should carry the drug home. That is one of the challenge and they say* – *they say we have not taken anything, as were coming to the health unit. That is where more of the challenge is.*

Health workers mentioned a range of other complaints they had heard from women attending ANC, including the size and number of the tablets, the unpleasant smell and bitter taste of SP. However, several health workers also pointed out that women can generally be persuaded to accept IPTp despite their concerns:

Health worker 5: *They don’t reject. They do it willingly. You just tell them* – *because once they come in contact with you, you are now friends. They know that* – *that is nurse, that is a medical worker. So now if I refuse to take this, they know it has no harm to me or to myself.*

The widespread positive views on IPTp and SP found in this research contradict frequent reports of negative perceptions including fear of side effects in the literature [42, 43]. Reluctance to take SP on an empty stomach has also been reported [37, 44], even though there is no evidence suggesting a link between nausea and taking SP on an empty stomach. From this research it appears that, in analogy with general concerns over taking medication in pregnancy, women can generally be persuaded to accept IPTp when recommended by a health worker. This has also been reported from a study conducted in Ghana, Kenya and Malawi [33].

## Conclusions

District health officials and health workers generally agreed that barriers relating to knowledge and attitudes towards ANC and IPTp among pregnant women and their communities accounted for many of the missed opportunities for the provision of IPTp. It was evident, however, that women and opinion leaders did not confirm district health officials’ and health workers’ suspicions. It is possible that district officials and health workers may have been inclined to ascribe low IPTp uptake to demand-side issues, rather than relating it to the supply side and hence their own work. Note that, in this study, it was not possible to include observation of interactions between health workers and women accessing ANC in order to triangulate the data obtained from interviews. Similarly, social desirability bias may have led some women and opinion leaders to express exclusively positive views of ANC and IPTp and not to elaborate on their concerns. However, there was no difference with regard to attitudes among women who were selected with the help of health workers and those selected by field researchers from ANC registers. There was also no substantial difference between women from the different regions and catchment areas included in the study, those who were pregnant at the time of the research and those who had given birth and between those living close to or far from the nearest health centre.

A potential limitation of the study is the fact that all women interviewed attended ANC regularly and the researchers found it challenging to identify women who had not received a full course of IPTp. It is therefore possible that the views of those identified may not fully reflect the reasons for late or infrequent ANC attendance and how this affects IPTp uptake. For example, some of the barriers discussed as challenges, but not ultimately reasons for non-attendance (such as burden of travel and financial burden of visiting ANC), may in fact play a greater role in deterring women from attending early or frequently than our data suggest.

As this research focused on barriers to IPTp uptake among women who attend ANC and deliberately excluded non-attenders of ANC, many of the barriers emphasized by district health officials and health workers and raised by many previous studies cited in the Results and discussion section (such as preference for traditional care or indifference towards preventive care) may act as significant barriers for those who do not attend ANC at all. However, our study did not intend to address these question. Similarly, this study intended to focus on public and PNFP facilities and therefore the lack of responses from clients accessing private care has meant that some challenges that may be specific to the private sector could not be explored in-depth. This appears to be particularly pertinent with regard to the issue of user fees and how they affect uptake of ANC and IPTp.

The generally positive views held by women and opinion leaders on ANC and IPTp, combined with the high ANC attendance figures, low refusal rates for IPTp and general trust in health workers’ judgement and advice, lead to the conclusion that, for those women who attend ANC, supply-side issues are likely to be more pertinent and to account for the majority of missed opportunities for the provision of IPTp. This is in line with a number of recent studies that have concluded that the main reason for not taking IPTp was not being offered the drug by the health worker [37, 44–47]. It has also been found that frequent ANC visits do not ensure access to IPTp-SP where other barriers exist [7, 46].

It appears likely that most women can be persuaded to accept IPTp and to return for subsequent doses if they receive encouragement and relevant information from health workers, especially where women report feeling dizzy or nauseous after receiving IPTp or where women express concerns about taking SP on an empty stomach. This suggests that greater impact on IPTp uptake could be achieved by intervening on the supply side (see Table 4 for a summary of the supply-side recommendations resulting from this study, including the document and record review [12]). However, the following actions should be considered by policy makers, implementers and researchers in order to strengthen demand-side use of ANC and acceptance of IPTp:

**Table 4:** Recommendations from study findings relating to the supply side

Stakeholders and coordination	Strengthen linkages between government and implementing partners
	Encourage vertical integration of health programmes, e.g. malaria and maternal and child health
Policies and guidelines	Ensure implementation of the most recent WHO policy recommendation of monthly IPTp administration after the first trimester
	Clarify policy of encouraging male involvement in ANC, ensuring women attending without their partners are not disadvantaged
Provision of ANC services	Ensure ANC is offered consistently in all health centres II, III, IV and hospitals as per national policy
	Encourage professional behaviour among health workers to create a positive ANC experience for all clients
	Provide incentives such as mosquito nets to encourage early and frequent ANC attendance
Supply chain	Consider supplying SP free-of-charge to private health facilities
Capacity building	Train health workers on malaria in pregnancy and IPTp to improve knowledge of the IPTp guidelines
	Consider use of alternative, non-disruptive training methods
	Provide guidance on differentiating between mild and severe side effects of SP and emphasize the importance of taking SP as directly observed therapy
Monitoring and evaluation	Ensure supply of standard recording and reporting tools to health facilities
	Ensure forms are designed to capture all plausible scenarios
	Improve health workers’ data management skills and provide clear guidelines with regard to recording conventions
	In addition to assessing completeness and timeliness of reported facility-level data, feedback should also be provided with regard to data accuracy

### Affordability

- Strengthen the supply chain of SP for private facilities, reducing the likelihood of private facilities charging for IPTp or asking women to buy SP from pharmacies or drug vendors.

### Acceptability

- Educate health workers and district officials with regard to ANC and IPTp uptake data from household surveys and HMIS, as well as research highlighting generally positive attitudes among women and communities to address the apparent misconception that demand-side barriers account for the majority of missed opportunities for the provision of IPTp.
- Design behaviour change messages, for example those delivered as part of routine health talks at health facilities, around the distinction between prevention and treatment, highlighting the preventive benefits of ANC and IPTp.
- Alert health workers to the role of trust in the relationship with their clients and their role in encouraging women to follow the recommended four-visit ANC schedule. Empower midwives to provide encouragement to pregnant women who have concerns over IPTp and SP to accept the medication, for example when women report having experienced mild side effects or do not want to take the drug on an empty stomach, insisting on providing IPTp as DOT at the health facility rather than letting women take the drug at home.
- Conduct research with women who do not attend ANC to determine barriers to ANC uptake among this group.

## Additional files

**Additional file 1.** Interview guide district health officials.

**Additional file 2.** Interview guide health workers.

**Additional file 3.** Interview guide women (English).

**Additional file 4.** Interview guide opinion leaders.

## Abbreviations

ANC: antenatal care
ANC1: first antenatal care visit
ANC4: fourth antenatal care visit
DOT: directly observed therapy (DOT)
HC: health centre
HIV: human immunodeficiency virus
HMIS: Health Management Information System
IPTp: intermittent preventive treatment in pregnancy
IPT1: first dose of IPTp
IPT2: second dose of IPTp
PNFP: private not-for-profit
SP: sulfadoxine-pyrimethamine
WHO: World Health Organization
